# Evaluation of an intragastric challenge model for *Shigella dysenteriae* 1 in rhesus monkeys (*Macaca mulatta*) for the pre-clinical assessment of *Shigella* vaccine formulations

**DOI:** 10.1111/apm.12168

**Published:** 2013-09-13

**Authors:** Dilara Islam, Nattaya Ruamsap, Patchariya Khantapura, Ajchara Aksomboon, Apichai Srijan, Boonchai Wongstitwilairoong, Ladaporn Bodhidatta, Montip Gettayacamin, Malabi M Venkatesan, Carl J Mason

**Affiliations:** 1 Department of Enteric Diseases, Armed Forces Research Institute of Medical Sciences (AFRIMS)Bangkok, Thailand; 2 Department of Veterinary Medicine, Armed Forces Research Institute of Medical Sciences (AFRIMS)Bangkok, Thailand; 3 Walter Reed Army Institute of ResearchSilver Spring, MD, USA

**Keywords:** Shigellosis, rhesus monkey (*Macaca mulatta*), challenge model

## Abstract

Shigellosis is a worldwide disease, characterized by abdominal pain, fever, vomiting, and the passage of blood- and mucus-streaked stools. Rhesus monkeys and other primates are the only animals that are naturally susceptible to shigellosis. A suitable animal model is required for the pre-clinical evaluation of vaccines candidates. In this study, the minimal dose of *Shigella dysenteriae*1 1617 strain required to produce dysentery in four of five (80% attack rate) monkeys using an escalating dose range for three groups [2 × 10^8^, 2 × 10^9^ and 2 × 10^10^ colony forming unit (CFU)] was determined. In addition, the monkeys were re-infected. The identified optimal challenge dose was 2 × 10^9^ CFU; this dose elicited 60% protection in monkeys when they were re-challenged with a one log higher dose (2 × 10^10^ CFU). The challenge dose, 2 × 10^10^ CFU, produced severe dysentery in all monkeys, with one monkey dying within 24 h, elicited 100% protection when re-challenged with the same dose. All monkeys exhibited immune responses. This study concludes that the rhesus monkey model closely mimics the disease and immune response seen in humans and is a suitable animal model for the pre-clinical evaluation of *Shigella* vaccine candidates. Prior infection with the 1617 strain can protect monkeys against subsequent re-challenges with homologous strains.

Severe acute *Shigella* dysentery is among the most miserable of human diseases. Shigellosis (bacillary dysentery) is an acute rectocolitis disease of humans and primates that is caused by invasion of *Shigella* in the large intestinal epithelium. It promotes a strong inflammatory response associated with fever, nausea, anorexia, dehydration, mucopurulent, bloody diarrhea and tenesmus 1,2. Worldwide, the number of cases of *Shigella* per year is an estimated 164.7 million, predominantly affecting populations in developing countries and children younger than 5 years of age 3. *Shigella* also remains a problem in travelers and military personnel deployed in developing countries 4,5.

*Shigella dysenteriae*1 (SD1), also known as *Shiga bacillus*, is the most virulent of the four serogroups of *Shigella* and is the only known cause of epidemic dysentery 6. Infection with SD1 causes a more severe and prolonged illness, with a much higher mortality rate than with other *Shigella* serogroups. SD1 disease contracted in the wild is notably resistant to most antibiotics commonly in use for the treatment of diarrhea 7. An effective vaccine is one that could reduce environmental risk from contaminated food and water, prevent the loss of combat effectiveness due to dysentery, and preclude the need for expensive new antimicrobials to treat multidrug-resistant species of SD1.

The rhesus monkey (*Macaca mulatta)* (RM) is the principal non-human primate species used in biomedical research 8. To assess the safety and efficacy of new vaccine candidates in the initial development phases, the pre-clinical evaluation first would be conducted in a reliable animal model prior advancement to clinical trials. To establish a relevant animal model that reproduces human bacillary dysentery, considerable effort has been devoted over the years to establishing a reliable animal model of bacillary dysentery, including a newborn mouse model 9, a guinea pig model 10,11, intestinal inflammation by ligated ileal loop assay in rabbits 12–14, and typical bacillary dysentery following intragastric inoculation in RMs 15–18. Humans, chimpanzees, and monkeys are the natural hosts for *Shigella* spp. 19, and the colon is the primary site of *Shigella* infection in humans 20.

In this study, we established the baseline of disease produced by the wild-type SD1 1617 strain in an RM model, which will serve as a reliable primate model for evaluating SD1 vaccine candidates. The challenge and re-challenge doses in this study were based on previous studies 17,18,21–24.

## Materials and Methods

### Experimental design

Fifteen RMs were randomly assigned to three dose-escalating groups of five monkeys each. In the initial challenge phase to identify the optimal dose, defined as the dose that produced dysentery (at least one watery or loose stool containing blood and mucus) in at least four of five monkeys (a ≥80% attack rate), the three groups were inoculated on study day 0 with target doses of 2 × 10^8^, 2 × 10^9^, and 2 × 10^10^ CFU of the SD1 1617 strain. The actual challenge and re-challenge doses are presented in Table 1. Thirty days following the initial challenge, the groups were re-challenged to test for the protectiveness of the prior infection with a dose that was one log higher than the initial dose, except for the last group, which was re-challenged with the same initial dose.

**Table 1:** Clinical signs of monkeys after challenged and re-challenged with different inoculum doses of SD1 1617 strain

Group	SD1 1617 inoculum dose CFU	Clinical signs	Number of affected monkeys	Antibiotic initiation day after challenge (number of monkey)
1 (n = 5)	Challenge: 2.08 **×** 10^8^	Dysentery	2	3 (1)
		Soft/normal stool with mucus and blood	3	
	Re-challenge: 2.02 **×** 10^9^	Dysentery^1^	1	NR
		Normal stool with mucus and blood	1	
2 (n = 5)	Challenge: 2.02 **×** 10^9^	Dysentery, one with vomiting	5	4 (2)
	Re-challenge: 1.84 **×** 10^10^	Loose/soft stool with mucus	2	NR
3 (n = 5)	Challenge: 1.76 **×** 10^10^	Dysentery, three with vomiting^2^	5	1 (2); 2 (1); 3 (1)
3 (n = 4)	Re-challenge: 1.6 **×** 10^10^	Nil	0	NR

NR, not required. Dysentery was defined as at least one watery or loose stool containing blood and mucus. Challenged on day 0 and re-challenge on day 31.

1 Same monkey that had dysentery after challenge.

2 One monkey died within 24 h.

All of the RMs used for this study were naïve to *Shigella* exposure by history (as determined by negative fecal cultures) and by *Shigella*-specific serum ELISA IgA and IgG titers <1:100. The monkeys were between 4 and 10 years old, weighed >5.5 kg, were of either sex, and came from the RM breeding colony at the Department of Veterinary Medicine, AFRIMS. The RMs of both sexes were determined to be healthy by physical examination and by normal values in blood cell counts.

Animal work was performed under a protocol approved by AFRIMS’s Institutional Animal Care and Use Committee. All of the described animal experimentation was in compliance with the Animal Welfare Act, adhered to the principles enunciated in the Guide for the Care and Use of Laboratory Animals, and was in accordance with all of the applicable guidelines of US Department of Agriculture, Office of Laboratory Animal Welfare, and Department of Defense. The Department of Veterinary Medicine, AFRIMS, is fully accredited by the Association for the Assessment and Accreditation of Laboratory Animal Care International, and the animals are cared for in accordance with the ‘Guide for the Care and Use of Laboratory Animals’, published by the National Research Council in 1996. All of the monkeys were observed by animal technicians certified by the American Association of Laboratory Animal Science. AFRIMS has maintained breeding colonies of RM for more than 33 years. Intensive veterinary care and preventive medicine are conducted to maintain the monkeys in healthy conditions.

### Inoculum preparation

The inoculum dose (both for challenge and re-challenge) was prepared from frozen vials from the master cell bank of the SD1 1617 strain. The bacteria were grown on a Congo red agar plate, and Congo red-positive colonies were inoculated into Luria-Bertani broth (LBB) and grown with shaking for 4–5 h. The LBB was prepared using yeast extract, tryptone (Becton-Dickinson, Sparks, MD, USA), and sodium chloride 25. The broth served as a starter culture for a large quantity of LBB, which was also grown with shaking for 4–6 h to obtain an OD_600_ of 0.5–0.6, (measured with a spectrophotometer, Biomate 3; Thermo Scientific, Madison, WI, USA) grown to log phase, when the bacteria were highly virulent (shown by plaque assay). The bacterial pellet was collected from the LBB by spinning and resuspended in PBS to obtain an OD_600_ of 0.9–1.0, which corresponded to approximately 1 × 10^9^ CFU. The method of preparation of the inoculum was the same for both the challenge and re-challenge phases. The actual challenge and re-challenge inoculum doses were measured by culture method, and their invasiveness was checked by plaque assay.

### Challenge and re-challenge protocol

All of the RMs were fasted overnight before challenge/re-challenge and for 90 min after challenge/re-challenge. On the challenge/re-challenge day, the monkeys were anesthetized with ketamine hydrochloride (10–20 mg/kg), administered intramuscularly in the caudal thigh. An 8 FR pediatric feeding tube 15 inches long was used for intragastric administration of the sodium bicarbonate buffer and inoculum. Twenty milliliters of sodium bicarbonate buffer solution (1.33%, pH 8.12) was administered intragastrically through the tube, and administration of the SD1 1617 challenge/re-challenge inoculum dose in 20 mL of sterile PBS followed. The monkeys were kept in an upright position for 10 min before they were returned to their individual cages.

### Illness index/monitoring of infection

A clinical scoring index was developed to assess the morbidity- and mortality-associated effects with the challenge dose in the monkeys, which was adopted from the ‘Guidelines on the Recognition of Pain’ 26. The parameters for clinical scoring (scored from 0 to 2/3, normal to severe) were (i) activity level, (ii) mentation, (iii) breathing, (iv) tremors, (v) vomiting, (vi) skin turgor, (vii) fecal consistency, (viii) fecal blood or mucus, and (ix) appetite. If the total score was ≤4, the monkey was in normal health; 5–9, the monkey needed to be monitored carefully and might be provided electrolytes in the drinking water; 10–14, the monkey had to be provided electrolytes in the drinking water or intra-venous fluid and analgesics were considered; and if score was ≥15**,** fluid therapy and antibiotics were started and the monkey was monitored closely every 2 h. Beginning on study day–3 (3 days prior to challenge) and continuing until the last study day, all of the monkeys were closely observed and scored twice daily using the clinical scoring sheet. All of the monkeys were monitored by direct observation for 30 min (after challenge/re-challenge) for the occurrence of immediate adverse reactions. IPTT-200 remote sensing transponder (BioMedic Data Systems, Inc., Seaford, DE, USA) thermometers were placed on the monkeys subcutaneously to measure the body temperature and temperature was recorded twice per day. Monkeys showing any signs of clinical disease were physically examined by a veterinarian and provided appropriate veterinary medical care according to the clinical scoring sheet. The antibiotic therapy administered was ciprofloxacin 16–20 mg/kg orally (PO) via naso-gastric tube, twice daily (bid) for 10 days. Antibiotic therapy was started on day 15 (if not required earlier) after the initial challenge and on day 46 (if not required earlier), 15 days after the re-challenge. Dysentery was defined as at least one watery or loose stool containing blood and mucus.

### Biosamples

Multiple blood samples, stool specimens, and colonic biopsies were obtained from each monkey.

### Blood

Blood samples were drawn from a superficial vein into vacutainer tubes containing Na-heparin or EDTA. Blood in EDTA tubes was used for assessment of a complete blood count (CBC), using the SYSMEX XT2000i (Sysmex America, Inc., Mundelein, IL, USA) automated hematology analyzer. CBCs were performed on each animal on days −17, −3, 4, 7, 14, 28, 35, 38, 45, and 59. Additional samples were submitted at the discretion of the attending veterinarian. Routine manual white blood cell differential counts were performed on Giemsa-stained blood smears. The blood in Na-heparin vacutainer tubes was separated into plasma and peripheral blood mononuclear cells (PBMCs).

### Stool

Stool specimens were collected from the cage tray of each monkey twice per day (in the morning and in the afternoon) for the detection of the challenge strain by standard culture procedures and by real-time PCR (RT-PCR) assay and for the measurement of fecal secretory IgA (s-IgA) and fecal cytokines. Immediately after collection, the stool samples were stored at −70 °C until extraction was performed. Stool samples were extracted with the extraction solution, as described earlier 27.

### Colonic biopsy

Colonic biopsies were obtained to measure histological changes 23,28. Colonic evacuation was performed on the day after the monkeys developed clinical signs of dysentery and a colonic biopsy was obtained on the following day. The RMs were pre-anesthetized with ketamine and maintained under anesthesia with isoflurane gas for the required procedure. Endoscopic biopsies were taken from the descending colon (12–22 cm from the anus) with an endoscope. Pinch biopsies (1–2 mm) were obtained from grossly normal rectal mucosa and from any abnormal mucosal lesions. Three colonic biopsies were obtained for routine microscopy from each animal on day −3 (which served as baseline), on the day after the monkeys developed clinical signs of dysentery after challenge/re-challenge. The biopsies were fixed in 10% formalin and processed for conventional histological evaluation using hematoxylin and eosin-stained sections for a descriptive morphologic diagnosis 23,29.

### Laboratory tests

#### Plaque assay

To assess the ability of SD1 1617 to invade epithelial cells and to grow intracellularly, the procedure for plaque assay, as developed by Dr. Edwin Oaks, was followed with minor modifications 30. Confluent LLC-MK2 (ATCC- CCL7; cell suspension prepared from adult rhesus monkey kidneys) monolayer cells were infected with the SD1 1617 strain. The uninfected, live cells were identified by uptake of the red dye, while the infected (dead) cells had no uptake and appeared as hollow plaques. Plaque assay was performed with both the challenge and re-challenge doses to confirm the invasiveness of the inoculum strain.

### Stool culture

The stool specimens were cultured on Hektoen enteric agar and Salmonella-Shigella agar plates and then placed in tubes of Kligler’s Iron Agar. Finally, they were identified by slide agglutination using commercially available SD1 antisera (Denka Seiken, Tokyo, Japan) to confirm the presence or absence of the SD1 strain.

### RT-PCR detection of SD1 in stool

DNA extraction from fecal samples was performed using the QIAamp Mini Stool Kit (Qaigen, Valencia, CA, USA), according to the manufacturer’s instruction manual. Purified DNA was used in the RT-PCR assay. Oligonucleotide sequences, derived from ipaH coding regions localized within a large plasmid of *Shigella flexneri* strain 5 (M90T), were used as detection primers, in addition to a TaqMan probe, as previously described 31–33. The Sequence Detection System 7700 (Applied Biosystems, Foster City, CA, USA) was employed for RT-PCR cycling amplification, real-time data collection, and analysis. The amplification data were collected and analyzed with Sequence Detection Software, version 1.9 (Applied Biosystems).

### Cellular immune responses

Pro-inflammatory cytokine levels were measured in plasma and in fecal extract samples using the FlowMetrix™ System (Luminex Corp., Austin, TX, USA) according to the manufacturer’s instruction, for the quantitative assay of IL-1β, TNFα, INFγ, IL-6, and IL-8 34,35.

### Antigens for immunological assays (ELISA and ELISPOT assays)

Immune responses were evaluated using the following antigens: SD1 LPS (Commonwealth Biotechnologies, Inc., Richmond, VA, USA), SD1 Invaplex protein antigens (Department of Enteric Infections, Walter Reed Army Institute of Research, USA), and BSA control antigens 36,37.

### Humoral immune responses

The systemic humoral immune response in plasma was evaluated using standard ELISA to detect IgA, IgG, and IgM antibody titers against SD1 LPS and SD1 Invaplex antigens 37. The net OD was equal to antigen-specific OD – BSA OD, OD measured with the SpectraMax® M2e Microplate Reader, (Molecular Devices, LLC, Sunnyvale, CA, USA). The end point titer for IgA was dilution showing a net OD of 0.10; the end point titer for IgG and IgM was a net OD of 0.20. Antibody titers were determined by four parameter analysis using Soft Max-Pro software, Molecular Devices, LLC. For the challenge phase, seroconversion was defined as a >4-fold rise in antibody titers on days 7, 14, and/or 28, compared with the baseline levels recorded on day −3. For the re-challenge, seroconversion was defined as a >4-fold rise in antibody titers on days 35, 38, 45, and/or 59, compared with the day 28 levels.

### Mucosal immune responses

The s-IgA response in the intestinal mucosa is a primary defense against enteric infections. SD1 LPS- and SD1 Invaplex-specific mucosal responses were determined indirectly by the measuring IgA, IgG, and IgM antibody-secreting cells (ASC) in circulation using ELISPOT assay 38.

Mucosal SD1 LPS- and SD1 Invaplex-specific s-IgA responses were determined in fecal extract samples using the ELISA procedure (mentioned above). After the challenge, a significant response was defined as a ≥4-fold rise in antibody titers compared with the baseline levels recorded on day −1; similarly, after the re-challenge, a significant response was defined as a ≥4-fold rise in antibody titers compared with the baseline levels recorded on day 30 37.

### Antibody-secreting cell assay

Enzyme-linked immunospot (ELISPOT) assay was performed to enumerate IgA, IgG, and IgM ASCs on fresh PBMC specimens against SD1 LPS and SD1 Invaplex antigens using Multiscreen Immobilon-P filtration, 96-well plates, EMD Millipore Corporation, Billerica, MA, USA 37. After the development of spots with substrate (NBT/BCIP), the ELISPOT plates were scanned on a CTL Immuno Spot Scanner/Analyzer, (CTL Worldwide, Shaker Heights, OH, USA) to count the spots, and this number was expressed as an ASC count per 10^6^ PBMCs. A positive (+ve) ASC response was defined as a count greater than the mean plus 3 SD [>(MN + 3SD)] of the baseline (day −3 or day 28) ASC count observed on day 7 after the challenge and on day 38 after the re-challenge, respectively. For baseline ASC counts that were equal to 0, a +ve ASC response was defined as an ASC count ≥10 ASCs per 10^6^ PBMCs on day 7 and on day 38.

### Data analysis

Spearman’s rank correlation coefficient (ρ) was computed to assess the relationship between the mean numbers of days the monkeys shed the challenge strain in their stool and the day on which the monkeys initiated antibiotic treatment. ELISA data (titers) were transformed into log scales and are presented as geometric mean titer (GMT) with (±) standard errors of the means (SE). Standard curves for the various cytokines (ranging from 0.25 to 1000 pg/mL) were constructed by a four parameter regression formula and plotted as a linear curve (log-log). The cytokine concentrations of the samples were calculated using ELISA Plus software, version 3.01 (Med Data Inc., Brecksville, OH, USA).

## Result

### Clinical outcomes

**Figure 1:**
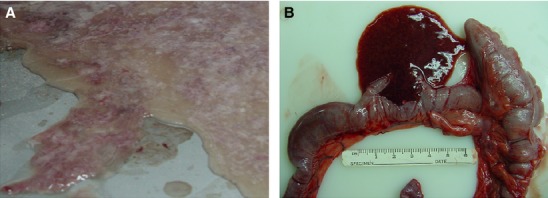
One monkey died within 24 h of challenge with a dose of 1.76 × 10^10^ CFU. (A) Inflamed intestine with only blood and mucus in feces, (B) bloody fluid in ileum.

The durations of GI disturbances, including the frequencies of clinical signs and symptoms following the challenge and re-challenge, in each group of monkeys are presented in Table 1. Forty percent of the monkeys in group 1 developed dysentery following the initial challenge with 2 × 10^8^ CFU. After re-challenge with 2 × 10^9^ CFU, one monkey developed dysentery (the same monkey that had dysentery after the challenge). The challenge dose, 2 × 10^9^ CFU, administered to group 2, met the definition of the optimal dose when five of the five monkeys (100%) developed dysentery, and two monkeys required early antibiotic treatment. Following re-challenge with 2 × 10^10^ CFU, two of five still developed clinical symptoms. The third group was challenged with 2 × 10^10^ CFU, which also resulted in an attack rate of 100%; all five monkeys developed severe dysentery. Twenty-four hours following the initial challenge, one of the monkeys died, and the remaining four monkeys required early antibiotic treatment. The monkey that died had inflamed intestines with bloody fluid, and only blood and mucus were found in the stool during autopsy (Fig. 1). Re-challenge in the remaining four monkeys showed that 100% of monkeys were protected, as none of them developed any significant clinical signs. The number of monkeys that required early antibiotic treatment also suggests the severity of the infection. Table 2 shows the average clinical score in each monkey group after challenge with SD1 1617 strain. Especially for days 1–3, the obtained score in each group was challenge dose-dependent and the score in group 3 was significantly higher than the score in both group 1 and group 2; however, there was no significant difference between the obtained scores between group 1 and group 2. For days 4–6, the average clinical score was still high in all three monkey groups and the score in group 3 was significantly higher than the score in group 1. For days 7–9, the scores in the group 1 monkeys had normalized, whereas monkeys in group 2 and group 3 still had high scores and group 2 had higher score than group 3 may be due to early antibiotic initiation in group 3. After re-challenge, the average score in each monkey group was <4, although one monkey in group 1 and one monkey in group 2 had score ≥5 for few days.

**Table 2:** Clinical score of monkeys after challenge with SD1 1617 strain

Study days	Average score in each monkey group (standard deviation)		
	Group 1 (10^8^ CFU)	Group 2 (10^9^ CFU)	Group 3 (10^10^ CFU)
(−3) to 0	2 (1)	1 (1)	1 (2)
1–3	3 (3)	6 (4)	12 (3)
4–6	6 (4)	7 (5)	10 (3)
7–9	4 (4)	7 (4)	5 (3)
10–15	2 (2)	3 (3)	2 (2)
After re-challenge	Normal (<4)	Normal (<4)	Normal (<4)

### Shedding of the SD1 1617 strain

The average number of days with ranges, each monkey group shed the SD1 1617 strain in their stool were recorded by both culture method and RT-PCR assay (Table 3) following the challenge and re-challenge. Early antibiotic treatment of monkeys, especially for the group 3 monkeys, might have reduced the shedding days detected by culture. However, RT-PCR could detect live or culture-negative *Shigella* in fecal samples, which indicates continued colonization of the lower gastrointestinal tract; hence, RT-PCR assay detected more shedding days compared with the culture method. The day on which the monkeys initiated antibiotic treatment was found to be correlated with the mean number of days of shedding by both RTPCR (ρ = 0.84, p < 0.001) and culture (ρ = 0.88, p < 0.001). One monkey in group 1 and two monkeys in group 3 did not shed the SD1 1617 strain as detected by culture method after re-challenge. Although both the group 2 and group 3 monkeys were re-challenged with similar doses, more monkeys in group 2 shed the strain compared with the group 3 monkeys, as the group 2 monkeys had GI disturbances after the re-challenge, and none of the group 3 monkeys had any sickness.

**Table 3:** Average number of days with *Shigella*-positive fecal samples after challenge and re-challenge with the SD1 1617 detected by standard culture method and real-time PCR (RT-PCR) assay

Group	Average number of days (range) with *Shigella*-positive fecal samples in each group			
	Post-challenge:		Post-re-challenge:	
	RT- PCR	Culture	RT- PCR	Culture
1 (n = 5)	13 (10–15)	6 (3–9)	13 (11–16)	4 (0–15)
2 (n = 5)	11 (8–13)	4 (3–7)	13 (12–14)	6 (1–8)
3 (n = 4)	7 (6–8)	2 (1–3)	13 (10–15)	2 (0–4)

Positive culture indicates excretion of viable *Shigella* bacteria. Positive RT-PCR indicates presence of culturable and non-culturable *Shigella*.

### Histological and hematological findings

Coarse (gross) observation was undertaken to view the histological changes in monkeys before and after challenge and re-challenge at the time of colonoscopy by the veterinary surgeon. Pre-challenge, before re-challenge and post-re-challenge, the histological features were within the normal limits. After the challenge, marked mucosal edema was observed in two of five monkeys in group 1, four of five monkeys in group 2, and three of four monkeys in group 3; the remainder of the monkeys had mild mucosal edema. Table 4 shows both the histological and hematological changes in the monkeys after the challenge and re-challenge.

**Table 4:** Histological and hematological changes in monkeys

Monkey group	Days	Histological changes		Hematological changes				
		Goblet cells decreased	Colonic acute inflammation	Lymphocytosis	Eosinophilia	Neutrophilia	Left shift	Toxic change
1	Post-challenge	1/5	1/5	0/5	0/5	2/5	4/5	4/5
	Post-re-challenge	0/5	0/5	0/5	0/5	1/5^1^	1/5^1^	1/5^1^
2	Post-challenge	4/5	1/5	0/5	0/5	2/5	5/5	5/5
	Post-re-challenge	1/5	1/5	4/5	4/5	0/5	5/5	3/5
3	Post-challenge	4/4	3/4: moderate necrotizing colitis 1/4: mild Acute inflammation	1/4	0/4	1/4	4/4	4/4
	Post-re-challenge	0/4	2/4: mild; 1/4: moderate eosinophilic inflammation 1/4: mild acute inflammation	1/4	4/4	0/4	1/4	1/4

Left shift (presence of immature neutrophils such as band cells in circulation) and toxic changes in neutrophils often observed as cytoplasmic Döhle bodies (retained aggregates of rough endoplasmic reticulum). These changes indicate an acute inflammatory response.

1 Same monkey.

#### Histological changes

Acute/diffuse colitis was observed (neutrophilic/necrotizing/hemorrhagic), with decreased goblet cells (indicating increased mucosal turnover), congestion, intraepithelial neutrophils, and crypt abscess, with epithelial attenuation and regeneration; these factors account for the acute inflammation and were observed in the RMs challenged with the SD1 1617 strain.

**Figure 2:**
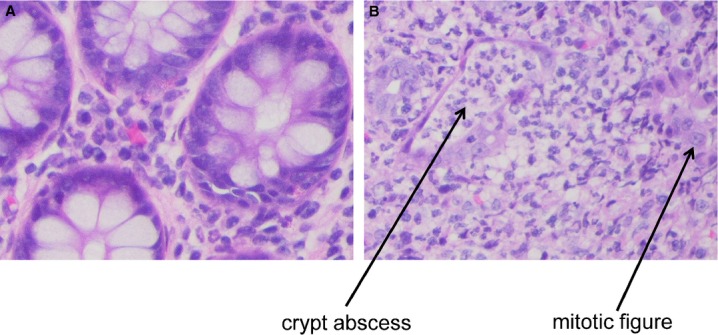
Histology of colonic biopsies collected before challenge (A) and 2 days post-challenge, (B) H&E-stained post-challenge biopsy shows acute neutrophilic colitis, with crypt abscess, increased numbers of neutrophils, decreased numbers of goblet cells, and an increase in mitotic figures.

**Figure 3:**
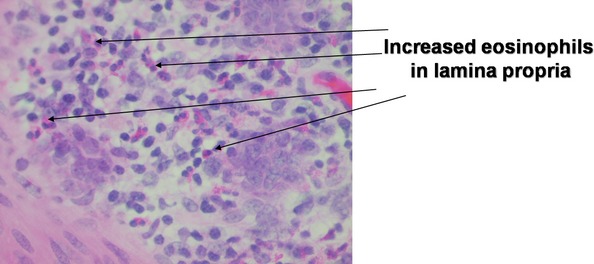
Moderate eosinophilic inflammation in the lamina propria.

In group 1, after the challenge, only one of five monkeys developed an appreciable increase in colonic acute inflammation. There were no changes in the colonic biopsies after the re-challenge. In group 2, after the challenge, four of five monkeys had decreased goblet cells, and one monkey had acute colonic inflammation. After the re-challenge, only one monkey had acute colonic inflammation. In group 3, after the challenge, all of the monkeys had severe dysentery, and one monkey died within 24 h. Of the remaining four monkeys, three monkeys were biopsied on day 2, and those three monkeys developed significant acute necrotizing colitis (Fig. 2B). From the fifth monkey, biopsies were collected on day 4, and this monkey had mild acute colonic inflammation. All four monkeys had decreased goblet cells. After the re-challenge, no lesions were noted in any of the monkeys; however, three of four monkeys had an appreciable increase in the number of eosinophils present in the lamina propria of the colonic biopsies (Fig. 3), and the fourth monkey had only mild acute colonic inflammation. H&E-stained colonic biopsies collected before challenge, with normal features are shown in Fig. 2A.

#### Hematologic changes

Changes in white blood cells were observed in RMs challenged with the SD1 1617 strain, including lymphocytosis, eosinophilia, neutrophilia (when the number of lymphocytes/eosinophils/neutrophil granulocytes in blood is high) with left shift (presence of immature neutrophils, such as band cells, in circulation) and toxic changes in neutrophils (often observed as cytoplasmic Döhle bodies: retained aggregates of rough endoplasmic reticulum), as shown in Table 4. These changes indicated an acute inflammatory response. In group 1, four of five monkeys developed toxic changes with left shift, and two of those also developed neutrophilia. Only one monkey responded to the re-challenge, developing neutrophilia with left shift and toxic changes. In group 2, all five monkeys developed toxic changes with left shift, and two of those also developed neutrophilia. After the re-challenge, all five monkeys responded with a left shift; in addition, four developed lymphocytosis and eosinophilia. In group 3, all surviving four monkeys developed a marked left shift with toxic changes after the challenge, and one monkey developed neutrophilia. However, after the re-challenge, all of the monkeys developed moderate eosinophilia, and one monkey had concurrent lymphocytosis with left shift and toxic changes.

### Plasma antibody responses

**Figure 4:**
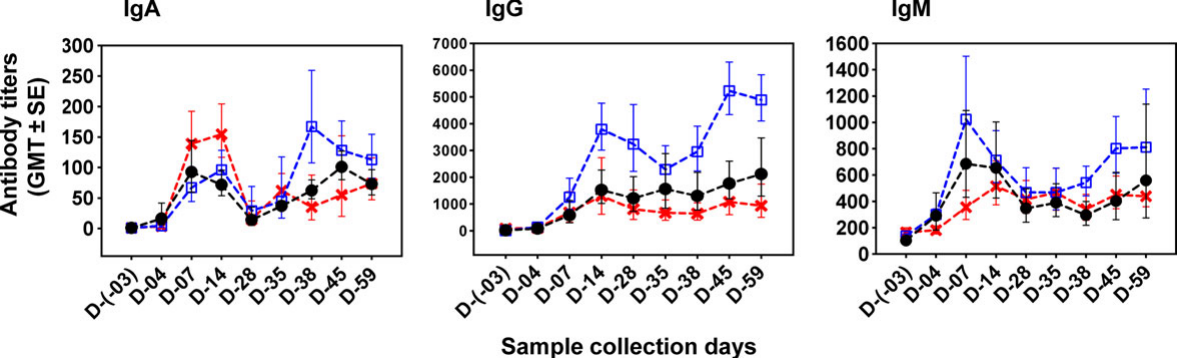
IgA, IgG, and IgM antibody titers against SD1-LPS in group 1 monkeys (cross symbol), group 2 (open square symbol), and group 3 (closed round symbol), expressed as geometric mean titer (GMT) with GMT±SE after challenge on day 00 and re-challenge on day 31 with SD11617 strain.

After the challenge, on both day 7 and day 14, all of the monkeys in all of the groups had seroconverted for IgA; all of the monkeys in group 2 and group 3 had seroconverted for IgG antibodies against SD1-LPS and SD1-Invaplex and for IgM against only SD1-Invaplex. After the re-challenge, only one to two monkeys in any group had seroconverted for IgA alone. In addition, among the antibodies measured, the largest response, in terms of changes in the magnitude of titers compared to baseline, was observed in IgG levels (Fig. 4). After the challenge, the IgA titers appeared to peak on either day 7 (Invaplex) or day 14 (LPS); the IgG titers appeared to peak on day 14 and the IgM titers appeared to peak on either day 7 or day 14. For group 1, group 2, and group 3, the mean peak fold increases as detected by serum were 40-, 3276-, and 221-fold for IgG-LPS assay and 126-, 8317-, and 6041-fold for IgG-Invaplex assay, respectively. As shown in Fig. 4, IgG-LPS levels rose steeply following the initial challenge between day 0 and day 14, dropped only slightly at day 28 and again rose steeply following the re-challenge to day 45, except for in group 3, in which the peak occurred on day 59.

### Antibody-secreting cell responses

Table 5 shows the SD1 LPS- and SD1 Invaplex-specific ASC counts in all the monkey groups after the challenge and re-challenge. The data are presented as geometric mean ASC counts with (±) SE. After the challenge, the IgA, IgG, and IgM ASC counts against LPS were highest in the group 1 monkeys, whereas after the re-challenge, the IgA, IgG, and IgM ASC counts against LPS were highest in the group 2 monkeys. After the challenge, the highest number of monkeys with +ve ASC responses to both antigens was in group 1 and after the re-challenge in group 2. Against SD1 LPS the +ve ASC response was IgM>IgG>IgA and against SD1-Invaplex IgA=IgG>IgM. After the re-challenge against both antigens, the +ve IgG-ASC response was low in all three groups. ASC counts specific for Invaplex were higher compared with the ASC counts specific for LPS. Similar to the plasma antibody titers, the ASC-Inv assays were more sensitive than the ASC-LPS assays.

**Table 5:** Antibody-secreting cells counts specific for SD1 LPS and SD1 Invaplex in each monkey group after challenge and re-challenge. Data are presented as GMT ± SE

	ASC counts per 10^6^ PBMC					
	Challenge (day 07)			Re-challenge (day 38)		
	Group 1 (n = 5)	Group 2 (n = 5)	Group 3 (n = 4)	Group 1 (n = 5)	Group 2 (n = 5)	Group 3 (n = 4)
ASC counts against SD1 LPS						
IgA	87 (36–207)	3 (2–8)	3 (2–6)	13 (6–27)	23 (10–51)	10 (2–43)
IgG	56 (25–123)	7 (3–16)	10 (4–25)	2 (1–6)	10 (5–19)	3 (1–11)
IgM	32 (21–48)	16 (8–34)	6 (3–10)	5 (2–9)	64 (47–88)	10 (4–23)
ASC counts against SD1 Invaplex						
IgA	323 (132–790)	310 (243–396)	116 (57–240)	17 (7–44)	267 (170–417)	225 (113–449)
IgG	262 (148–464)	299 (269–333)	183 (126–266)	29 (12–72)	196 (131–292)	45 (12–160)
IgM	43 (30–60)	7 (3–17)	7 (2–20)	2 (1–3)	61 (48–78)	5 (2–15)

### Fecal s-IgA antibody response

**Figure 5:**
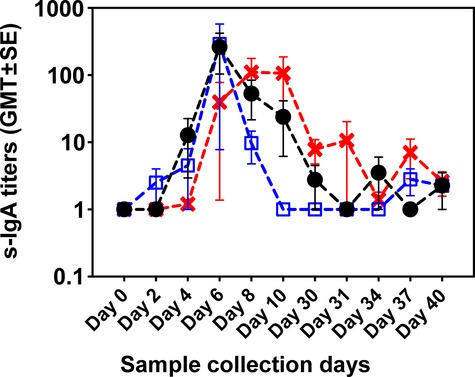
Fecal s-IgA antibody titers to SD1-LPS in group1 (cross symbol), group 2 (open square symbol), and group 3 (closed round symbol) monkeys, expressed as MN with MN ± SE, after challenge on day 00 and re-challenge on day 31 with SD11617 strain.

No dose-dependent fecal s-IgA responses were observed in the challenge groups. Two monkeys in group 1 and one monkey in group 2 had no detectable fecal s-IgA. Fecal s-IgA peaked on day 6 for both group 2 (peaked titer 90) and group 3 (peaked titer 261), but was delayed in group 1, peaking on day 8 (peaked titer 110). Several monkeys in all three groups had significant fecal s-IgA responses after the challenge for 5–10 days. Fig. 5 shows the s-IgA titer expressed as GMTs with (±) SEs in the three monkey groups over the study period.

### Cytokine responses

**Figure 6:**
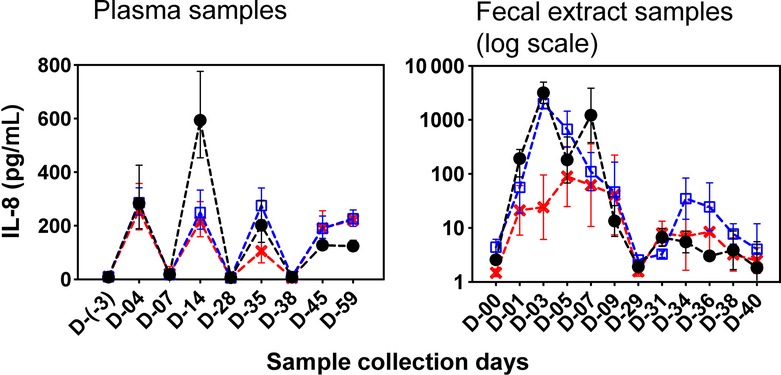
Cytokine IL-8 levels (pg/ml), in plasma (A) and in fecal extract samples, (B) in monkeys from group1 (cross symbol), group 2 (open square symbol), and group 3 (closed round symbol) after challenge on day 00 and re-challenge on day 31 with SD11617 strain.

Quantitative measurement of pro-inflammatory cytokines (IL-1β, TNFα, INFγ, IL-6 and IL-8) were performed on both the plasma and fecal extract samples. There were no significant cytokine responses in plasma samples. Figure 6 shows IL-8 levels (pg/mL) in plasma samples and fecal extract samples. Among five cytokines, only IL-1β and IL-8 cytokine responses were detected in fecal extract samples. All three groups showed evidence of dose-dependent response in fecal IL-1β and IL-8 cytokine levels on days 3–9 following the challenge. On study day 5, the cytokine responses were high in group 2 monkeys, perhaps due to early antibiotic treatment initiation in group 3 monkeys, the IL-8 response was low. After re-challenge, only in group 2 monkeys, cytokine responses were seen; however, the level was low.

## Discussion

In this study, we evaluated intragastric challenge model for *S. dysenteriae* 1 (SD1SD1) in rhesus monkeys for the pre-clinical evaluation of SD1 vaccine candidates. Although numerous studies in RMs challenged with *Shigella* have been reported earlier, a recent evaluation of this model was necessary before vaccine evaluation. With new SD1 vaccine candidates in the pipeline, a suitable animal model, which mimicked infection in humans, would facilitate the pre-clinical evaluation of vaccine candidates’ advancement to clinical trials. Although the infective dose in humans is only 10–100 bacteria 21, it was shown earlier with a *S. flexneri* strain that the corresponding dose in monkeys was 10^9^–10^11^ bacteria 39. In this study, we showed that the infective dose (100% attack rate) of the SD1 1617 strain in adult rhesus monkeys is 10^9^ bacteria and that prior infection with the SD1 1617 strain can protect monkeys against subsequent re-challenges with homologous strain.

The more severe the initial infection is, the better the protection against re-infection is. Compared with our optimal dose for dysentery, which is 2 × 10^9^ CFU, in a recent study in Cynomolgus macaques, it required 10^11^ CFU to develop dysentery 40. One reason for the lower CFU requirement for the current study could be the use of log-phased bacterial culture to prepare inoculum dose. Log-phased broth culture was observed to be more virulent compared with overnight agar plate culture (data not shown).

The ability of *Shigella* to cause diarrheal illness is restricted to human and non-human primate hosts. Recently, a number of studies have been performed using the guinea pig model; although not an intestinal model, the guinea pig model was useful only for testing the efficacy of vaccine candidates and correlating efficacy with antibody responses. In a recent guinea pig model, typical bacillary dysentery occurred in animals challenged intrarectally, rather than by the natural oral route, with high doses of virulent *S. flexneri* strains 41. In another study, guinea pigs were infected with SD1 1 and *S. flexneri* 2a into the cecocolic junction, All the guinea pigs developed typical dysentery within 24-h post infection; in addition, orally immunized animals conferred homologous protective immunity against subsequent challenges with the live strains 14. Although these guinea pig model and rabbit ileal loop model 13 are useful to study pathogenesis and inflammatory responses, the artificial nature of these systems might hamper their ability to advance vaccine development in humans. To date, macaques remain the only animals in which typical bacillary dysentery can be induced by oral administration of *Shigella*.

The peripheral blood leukocyte and neutrophil counts are indicators of an immunoreactive event. In the current study, detailed histological and hematological analyses in monkeys with shigellosis were similar to those observed in humans with shigellosis 42–44. Previous studies have indicated that wild-type *Shigella* infection results in massive influx of neutrophils and monocytes to the infected portion of the intestinal mucosa 45. In this study, even the lowest challenge dose elicited 80% hematological changes (toxic changes and left shift, which may indicate an acute inflammatory response). The hematological changes in monkeys challenged with 2 × 10^9^ CFU of SD1 were more prominent compared with the histological changes, as after the re-challenge, 100% of the monkeys still showed toxic changes, whereas only 20% of the monkeys showed histological changes. These findings indicate that s-IgA excreted from the gut (mounted after the initial infection) might protect the gut mucosa, whereas *Shigella*-specific memory cells might not protect against systemic inflammation. Both mucosal s-IgA and serum IgG specific for *Shigella*-LPS, the major bacterial surface antigen, are elicited upon natural infection 42,46. Recent evidence indicates that they are the main factors in protection against re-infection 47 and that s-IgA preserves intestinal barrier integrity by preventing bacterial-induced inflammation 48. Exposure of RMs to SD1 1617 elicited the antibody responses to both LPS and invaplex protein antigens, although within a 1-month period, the antibody levels decreased to almost baseline levels, except IgG. The antibody response was dependent not only on the dose but also on the duration of exposure to the dose, as, group 2 monkeys had highest antibody responses, although group 3 dose was higher but got earlier antibiotic treatment. In addition, after re-challenge group 2 monkeys showed highest antibody responses.

Earlier, multiple cytokines were shown to be secreted in the blood and excreted in fecal samples in adults with shigellosis; moreover, the cytokine levels were correlated with the severity of disease 49. In plasma and in fecal extract samples in these challenged monkeys, we did not find a number of proinflammatory cytokines, like adults; only IL-1β and IL-8 were found in fecal extract samples, and this is the only discrepancy found in rhesus monkeys. The cytokine levels observed correlated with disease severity.

Both mast cells and eosinophils are resident in the gastrointestinal mucosa, and they can modulate both the innate and antigen-specific immune responses. Increased numbers of eosinophils and mast cells have been implicated in inflammatory, in allergic diseases, in parasitic infections, and in chronic inflammatory processes 50,51; however, it was shown earlier that in acute shigellosis, mast cells and eosinophils were upregulated in the gastrointestinal mucosa and that the response was prolonged and persistent, both in the peripheral blood and in the rectal tissue 52. There have been longer durations of illness in patients with shigellosis, and cellular organelle damage has been greater in the deeper layers of the crypt, with degranulation of eosinophils and mast cells and activated lymphocytes found in such patients 53. Similarly, in this animal model, we observed that in severe shigellosis, there is upregulation of eosinophils, both in the peripheral blood and in the colonic tissue, which indicates that the rhesus monkey model is a true copy of shigellosis in humans. The disease mechanism process is similar in human and in rhesus monkeys.

Earlier, the site and mechanism of fluid loss in shigellosis were studied 54 in monkeys, and it was found that dysentery results from a colonic transport defect, while diarrhea is secondary to jejunal secretion superimposed on the defect in colonic absorption. In the jejunum or ileum, however, morphological changes were minimal and bacterial invasion was not seen. Another study revealed that at necropsy of Shigella-infected monkeys, gross abnormalities were confined to the colon. The small intestine appeared normal except for the terminal ileum in some cases 24. Postmortem studies in children 55 and adults 44 with shigellosis showed that the most common underlying cause of death was severe colitis in large segments of jejunum and ileum, which was often complicated by septicemia, malnutrition, and pneumonia. Similar findings were obtained from the monkey that died in which the whole ilium was with bloody fluid.

Moreover, our findings suggest that, in addition to the standard culture method for the detection of *Shigella* in fecal samples, RT-PCR assay should be performed to identify colonization of *Shigella*, which is consistent with previous findings that have documented the limitations of traditional culture methods 56.

In conclusion, our findings confirm that the rhesus monkey model closely mimics the disease, immune response, and inflammatory responses seen in humans.

In the current study, we have presented evidence that rhesus monkeys are a suitable species to be used as a challenge model with wild-type SD1; therefore this animal model can be used in vaccine development and evaluation. However, the similarities in clinical symptoms, shedding of the organism, immune response, hematological and histopathologic findings between rhesus monkeys and humans infected with *Shigella* spp. makes this animal model the best currently available for studying pathogenesis, infection-derived immunity and, likely, vaccine efficacy.
